# Optimal Timing for Expanding Diagnostic Laboratories, South Korea

**DOI:** 10.3201/eid3108.241745

**Published:** 2025-08

**Authors:** Jae-Sun Park, Gab Jung Kim, Sang Won Lee

**Affiliations:** Korea Disease Control and Prevention Agency, Cheongju, South Korea

**Keywords:** communicable diseases, infectious diseases, COVID-19, respiratory infections, severe acute respiratory syndrome coronavirus 2, SARS-CoV-2, SARS, coronavirus disease, coronavirus infection, Middle East respiratory syndrome, MERS, diagnostic laboratories, zoonoses, viruses, South Korea

## Abstract

The rapid expansion of testing capacity is imperative for an adequate response to infectious diseases, such as COVID-19. South Korea rapidly secured large-scale testing during the early stages of COVID-19 in 2020 by leveraging the country’s experience with the 2015 Middle East respiratory syndrome outbreak; the initial response was relatively successful. A key difference between the 2 outbreak responses was the expansion from public to private testing laboratories during the COVID-19 pandemic. Expanding testing capacity during an infectious disease crisis should involve consideration of the overall response system and social conditions and not just the number of patients. If there are concerns about a crisis developing, testing capacity expansion should begin as soon as possible. Furthermore, accuracy should be ensured, especially when testing capacity is expanded. South Korea’s experience in developing diagnostic systems and adopting testing strategies underscores the value of proactive and well-timed preparedness for emerging infection disease outbreaks.

After the COVID-19 pandemic, securing the appropriate diagnostic reagents necessary for testing and expanding laboratories to increase testing access brought to the forefront the key competencies needed to respond to large-scale infectious disease epidemics. The 100 Days Mission to respond to future pandemic threats reported to the Group of Seven countries in June 2021 indicates that it took 64 days to announce the first Emergency Use Listing for PCR after the World Health Organization (WHO) declared a COVID-19 Public Health Emergency of International Concern (PHEIC) (*1*). In addition, insufficient numbers of molecular diagnostic laboratories and low manufacturing capacity of diagnostic reagents were cited as reasons for the lack of preparation for infectious disease outbreaks (*1*). When WHO announced the Emergency Use Listing for the COVID-19 real-time reverse transcription PCR reagent (April 3, 2020), the first COVID-19 wave that had centered in the Daegu region of South Korea had already passed. Several countries had reached the peak of the first epidemic in Europe and America, and >1 million cases of COVID-19 had been confirmed globally (*2*,*3*). Therefore, multiple countries faced the first wave without sufficient information regarding appropriate diagnostic reagents and laboratories for COVID-19 testing.

South Korea secured a large-scale testing capacity for COVID-19 more rapidly than other countries and used it for control measures. The Korea Disease Control and Prevention Agency (KDCA) determined that the coronavirus outbreak in China during December 2019 could spread to South Korea at any time because of the close proximity to China and active exchanges between the countries. Therefore, KDCA quickly established a method to begin testing for suspected COVID-19 in patients. After identifying an influx of cases in South Korea, we secured the diagnostic reagents and laboratories necessary for large-scale testing. A large-scale testing capacity of ≈20,000 cases/day was secured at that time, which continuously increased to ≈850,000 cases/day by February 2022. By rapidly identifying infected persons through extensive testing and then isolating those patients, we delayed the spread of infection until vaccines and medicines were introduced. Consequently, we maintained low mortality and severity rates and reduced social chaos and damage. One of the major factors enabling us to secure large-scale testing capacity was the lesson learned from the 2015 Middle East respiratory syndrome (MERS) outbreak.

## Comparison of MERS in 2015 and COVID-19 in 2020

### Laboratory Expansion in Response to MERS

**Figure 1:**
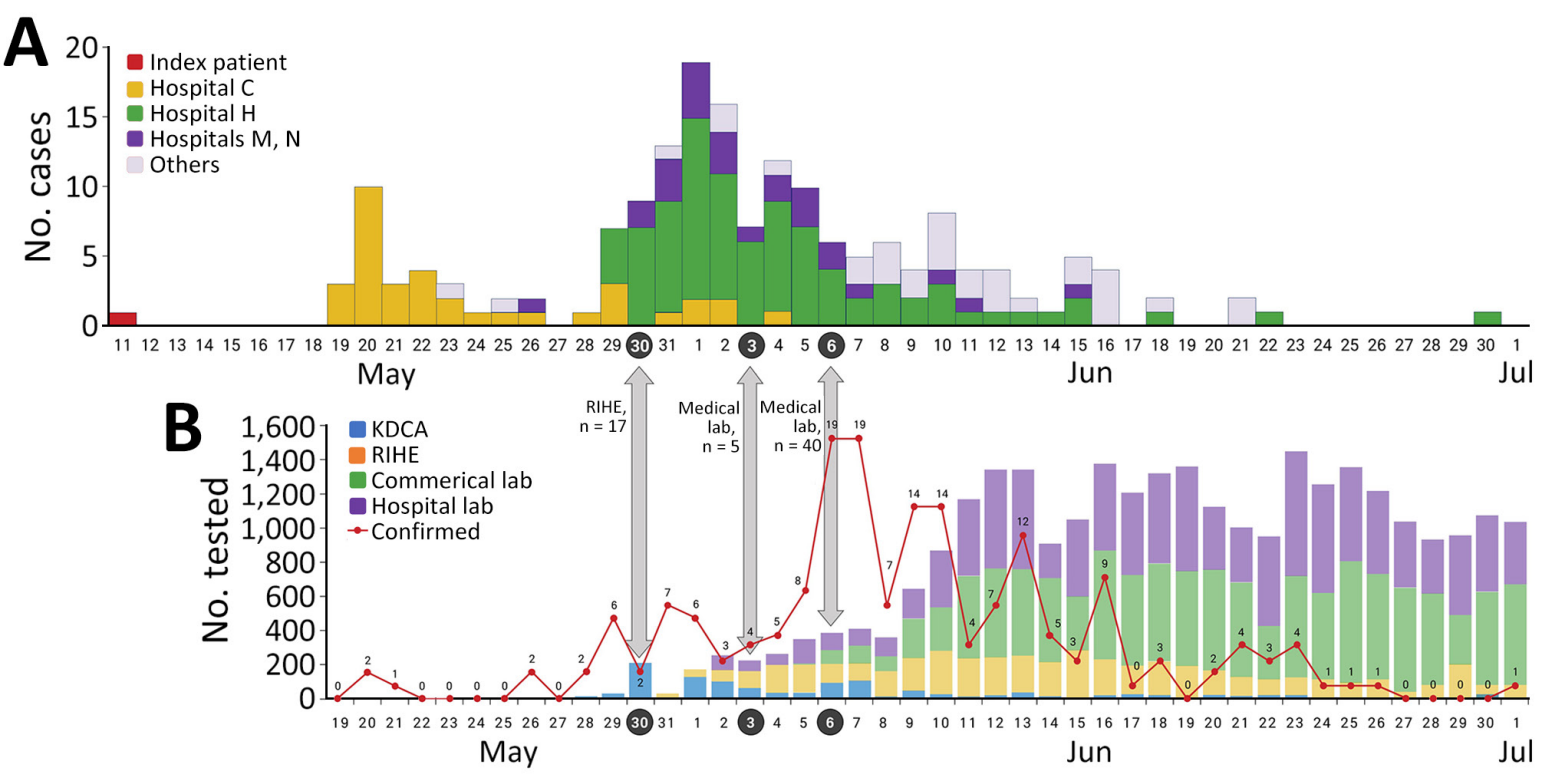
Epidemiologic curve of Middle East respiratory syndrome (MERS) cases and tests in South Korea during May–July 2015. A) Epidemiologic curve according to date of MERS symptom onset in patients, adapted from KDCA report (*4*). B) Number of daily tests for MERS, adapted from Ministry of Health and Welfare white paper (*5*). Gray arrows indicate expansion dates of new testing facilities. Red line with numbers indicates newly positive laboratory results. KDCA, Korea Centers for Disease Control and Prevention; lab, laboratory; RIHE, Research Institute of Health and Environment.

The number of testing laboratories expanded after the spread of MERS virus infections in 2015 (Figure 1). KCDA conducted all tests for MERS after the first case was detected on May 11, 2015, until the second wave began during late May because of in-hospital infection. On May 30, the number of laboratories expanded to include 17 Research Institute of Health and Environment (RIHE) centers belonging to local governments. As the number of patients continued to increase, testing expanded to include 5 commercial laboratories at medical institutions on June 3 and 40 hospitals on June 6. The number of cases declined after the peak of the MERS pandemic (*5*). The increase or decrease in the number of tests generally follows the pattern of increase or decrease in the number of patients. However, the number of MERS tests increased even though the number of patients decreased in early June 2015, because the testing capacity did not meet the testing requirements. Limited laboratory capacity might have caused delays in testing, leading to a gap between symptom onset and confirmation of results. It can be challenging to determine whether the demand for testing increases when the numbers of cases decrease. Instead, testing was tailored to the needs of the field and was established by using the necessary testing capabilities. In addition, no diagnostic reagents approved by the Ministry of Food and Drug Safety (MFDS) were available. Therefore, medical institutions had no choice but to use research-use only (RUO) products, which was an obstacle to rapid expansion of testing capabilities within private commercial laboratories and hospitals. It took time to determine whether RUOs could be used for testing and to establish which RUO products could be accurately and safely used for testing. Thus, the MERS outbreak experience highlighted that the demand for testing for respiratory infectious diseases could increase more rapidly than expected, that private testing capabilities must be actively used, and that active government leadership and appropriate systems are essential for using private testing laboratories.

Since the MERS outbreak, KDCA has formed a public–private consultative body to promote close cooperation with private experts and has established an emergency use authorization (EUA) system along with the MFDS for diagnostic reagents (*6*). The COVID-19 pandemic occurred after those efforts had been made; therefore, we quickly secured large-scale testing capacity during the early stages of the COVID-19 pandemic. The trust relationship built through the public–private consultative body has become an essential foundation for public–private partnerships responding to COVID-19. An EUA enables the temporary use of reagents that are not licensed for in vitro diagnostic medical devices, such as RUOs, to respond to infectious disease crises occurring after the MERS outbreak. The EUA system was first used in South Korea in 2016 after the WHO declared a Zika virus PHEIC (*6*). Diagnostic reagents developed by various manufacturers quickly entered the market through emergency approval, and sufficient reagents required for large-scale inspections were secured.

### Laboratory Expansion in Response to COVID-19

**Figure 2:**
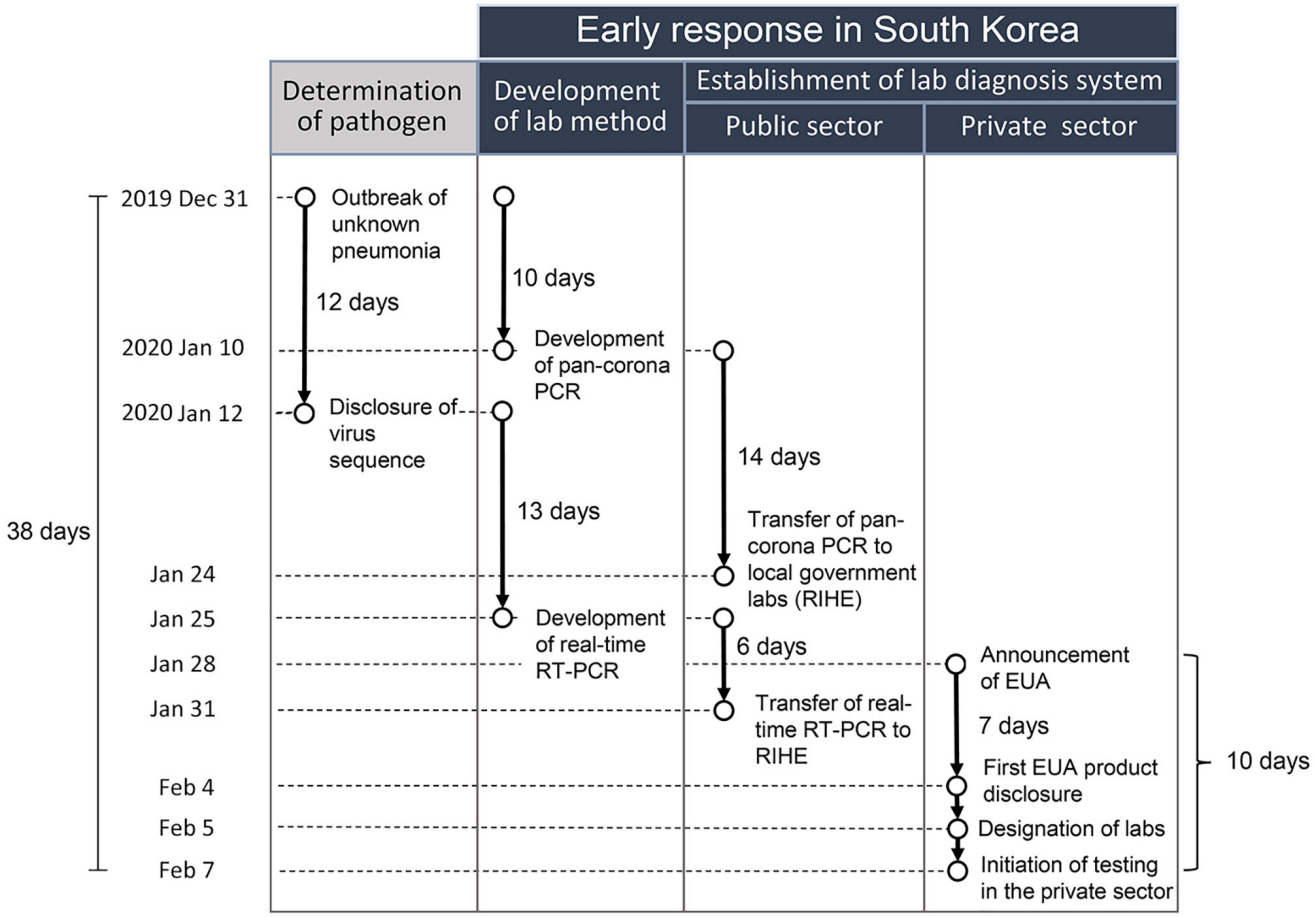
Flowchart of early diagnostic laboratory response to COVID-19 in South Korea in 2020. EUA, emergency use authorization; lab, laboratory; RIHE, Research Institute of Health and Environments; RT-PCR, reverse transcription PCR.

**Figure 3:**
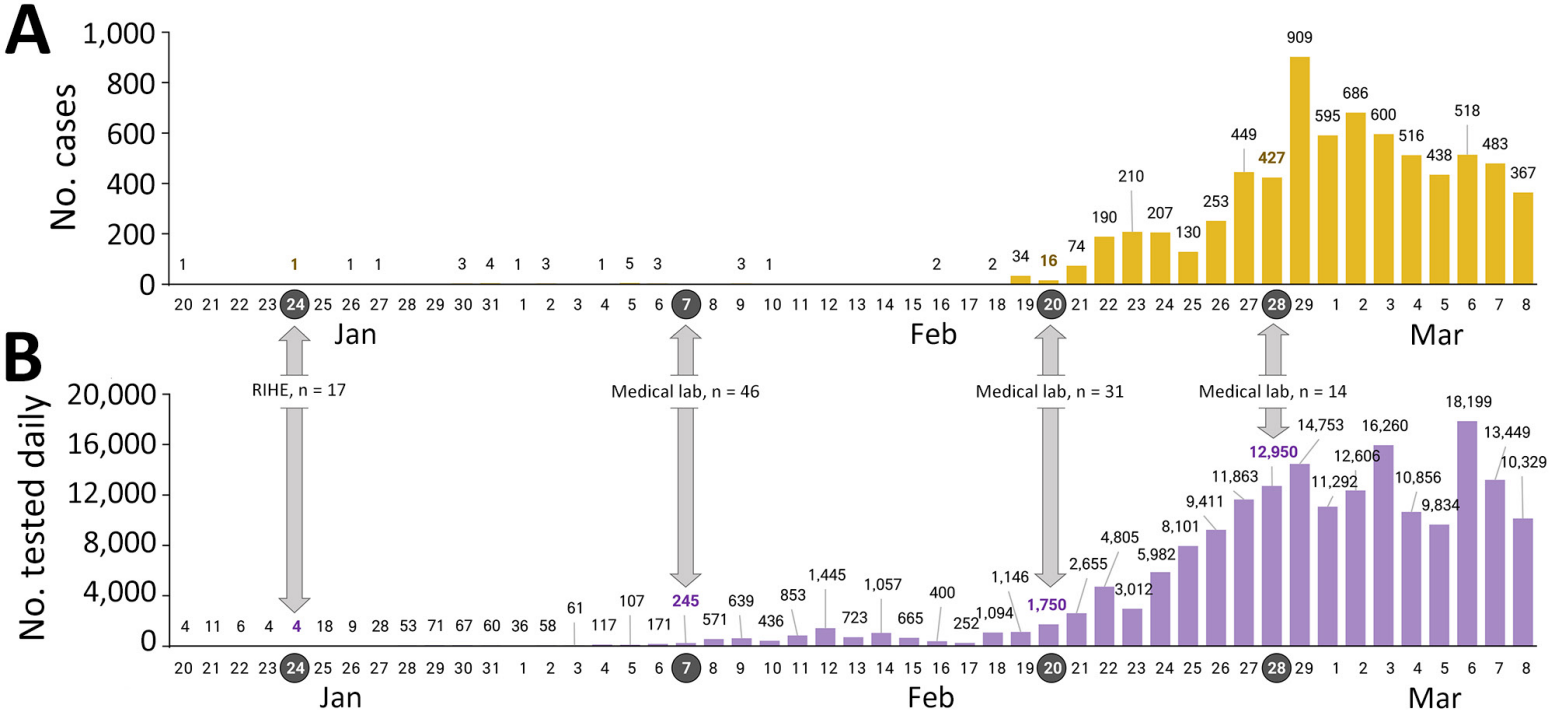
Numbers of COVID-19 cases and tests in South Korea during January–March 2020. A) Number of newly confirmed cases. B) Number of daily tests. Gray arrows indicate the dates (and numbers) private medical laboratories were integrated into the diagnostic laboratory system. Numbers above bars indicate actual numbers of cases or tests. Lab, laboratory.

During the early stages of the COVID-19 response, after the first case occurred in South Korea on January 20, 2020, and the second case occurred on January 24, 2020, KDCA immediately transferred testing technology to RIHE centers across the country. Accordingly, a nationwide testing network was established to test up to 2,000 persons per day. Subsequently, KDCA decided to expand testing capacity through laboratories at medical institutions when a total of 4 patients had a COVID-19 diagnosis, judging that a high risk of patient transmission of disease was possible because of the nature of the respiratory virus. MFDS-approved reagents are required to conduct tests at private medical institutions, but an EUA was pursued because no initially approved reagents for new infectious diseases, such as COVID-19, existed. To select products that could be used in emergencies, KDCA posted an evaluation notice for the EUA on January 28, 2020 (Figure 2). On January 29, KDCA disclosed its COVID-19 testing method so that reagent manufacturers could use it as a reference for developing products. The EUA process first involved the MFDS and the KDCA reviewing documents submitted by each manufacturer, and only products that had appropriate performance testing completed were evaluated against products from KDCA and the Korean Society of Laboratory Medicine (KSLM). The evaluation was conducted by determining whether the test performance was equal to or higher than that of the KDCA test method. KDCA requested EUAs from MFDS for products that had performance levels equal to or higher than those from the evaluation agencies. MFDS granted the first EUA on February 4, 2020, for a COVID-19 diagnostic reagent (Figure 2). In addition, we recruited medical institutions that wished to conduct COVID-19 testing, provided education on COVID-19 testing through the KSLM, and conducted an external quality assessment along with the Korean Association of External Quality Assessment Service. KDCA designated institutions that had completed training and passed proficiency tests as COVID-19 testing institutions. Therefore, on February 7, 2020, a total of 47 medical institutions completed training and proficiency tests and began testing for COVID-19 by using EUA products (Figure 2). The nationwide testing capacity has increased from 2,000 to 20,000 per day. As testing capabilities expanded, the actual frequency of testing also increased. Subsequently, the 31st case related to Shincheonji Church in Daegu was reported on February 18. On February 20, COVID-19 testing of ≈9,000 Shincheonji believers in Daegu began, which represented an increase in the number of medical institutions capable of testing for COVID-19 by 31 (Figure 3) (*7*). That addition enabled the country to meet the growing testing needs quickly and reliably with sufficient capacity. All tests were conducted by using EUA products evaluated by KDCA and KSLM, minimizing anxiety and confusion about the performance of the products and enabling more active testing in the private sector. In addition, KDCA and KSLM jointly published guidelines for testing, supporting safer and more accurate testing in the field.

**Figure 4:**
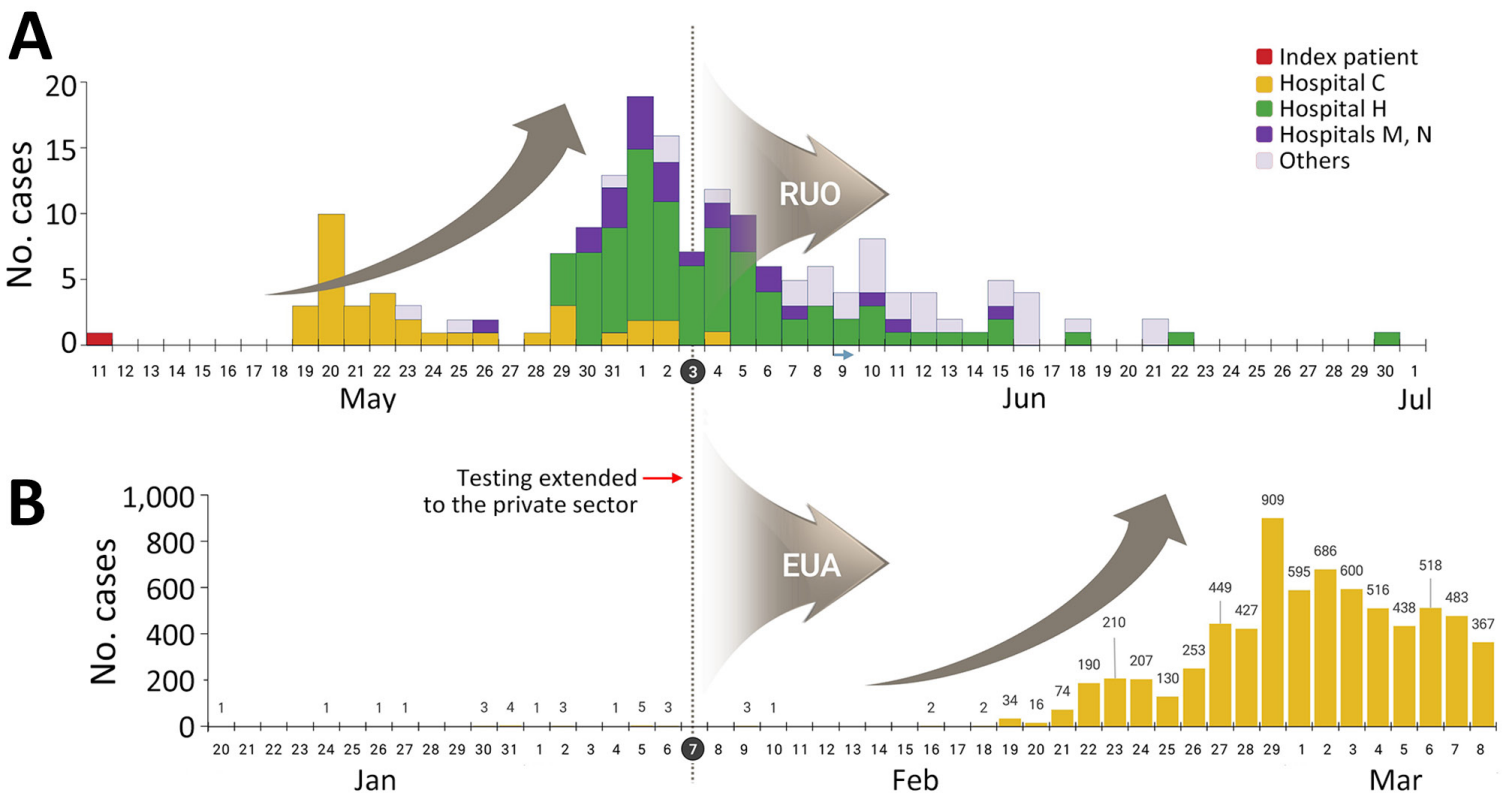
Diagnostic laboratory expansion for Middle East respiratory syndrome (MERS) and COVID-19, South Korea. Number of MERS cases during May–July 2015 (A) and number of COVID-19 cases during January–March 2020 (B) were compared. Red arrow and dotted vertical lines indicate when testing was expanded to include private medical testing laboratories. Gray curved arrows indicate when testing was conducted using RUO-approved or EUA-approved reagents. RUOs were used to implement new MERS diagnostic tests, and EUAs were used for novel COVID-19 diagnostic tests. EUA, emergency use authorization; RUO, research-use only.

On the basis of the systems and efforts established because of the MERS outbreak in 2015, South Korea rapidly and systematically secured the initial diagnostic testing capabilities for COVID-19. The difference between the responses to MERS and COVID-19 can be observed in the timing of testing capacity expansion that included the private sector (Figure 4), which indicates the necessity of securing sufficient and stable testing capacity before an outbreak begins. However, it is difficult to establish the timing for expanding testing capacity by predicting the spread of new pathogens. Moreover, excessive expansion of testing capacity for new pathogens, which might be accompanied by inaccurate information and a lack of skilled personnel and reagents with guaranteed performance, can lead to increased uncertainty because of incorrect test results. That uncertainty might lead to confusion and anxiety during infectious disease responses. Therefore, a new infectious disease in a country does not necessitate the expansion of its testing capacity. If it is possible to respond with a stable and verified system, testing should be conducted by using that stabilized system rather than unreasonably expanding testing capabilities. In contrast, countries should not hesitate to expand testing capacity because outbreaks are small. It is easier to treat and manage patients when tests are more accessible. The critical question is when the risk of using reagents in the field should be considered if their performance has not yet been fully verified (i.e., at what point do the benefits of expanding testing capacity outside the routine system outweigh the risks?).

## Concerns and Considerations for Expanding Laboratories

Testing capabilities should be expanded when a crisis caused by a new pathogen is expected. However, the incidence of cases does not necessarily indicate a crisis. The word crisis is used when the social effects of an infectious disease are considerable and cannot be controlled or when an uncontrollable situation is expected to occur. Problems arise because procedures in daily life do not provide the necessary diagnostic testing capabilities for responding to infectious diseases; COVID-19 is a representative example of that type of disease. A newly identified virus caused COVID-19, clinical trials to test reagents and the general approval process had not been completed, and experience testing at medical institutions did not exist. However, the demand for tests was predicted to increase markedly because of the virus transmission rate, mortality rate, and fears of a new virus. The experience with mpox was similar; approved reagents and testing experience were lacking. However, it was expected that the social effects of mpox and the rate of increase in testing demand would not be the same as that observed for COVID-19. Testing for mpox centered on a specific group; monkeypox virus transmission occurred through sexual contact and close physical contact in shared living environments. In addition, the mpox mortality rate was lower than that of COVID-19. Therefore, government agencies (KDCA and RIHE centers) conduct tests for mpox as part of the routine infectious disease response system. The effectiveness of those mpox test products has not been verified through MFDS; however, tests are being conducted with high accuracy by well-trained government personnel who use methods developed and verified in-house by KDCA. If an approved product for mpox testing is released in the future, general medical institutions might conduct testing; however, we did not expect an emergency or crisis large enough to expand the mpox testing capacity by temporarily using unlicensed reagents.

Crises do not necessarily occur because of the emergence of new pathogens. Infectious diseases that have previously occurred in small numbers in a country might become crises because of large-scale outbreaks or if genetic mutations occur in the pathogen. A crisis caused by a sudden large-scale outbreak of infectious disease might lead to a reduction of domestically secured diagnostic testing capabilities, or the use of existing diagnostic reagents might become impossible because of pathogen mutations. Therefore, it is challenging to delineate an infectious disease crisis that requires expansion of testing capacity according to a fixed framework, such as when more than a few cases are reported or when a specific pathogen is responsible. A crisis or its risks should be judged by considering professional opinions and the status of domestic professional infrastructure, pathogen characteristics, and experience in responding to infectious diseases, including public sentiments. In particular, with regard to professional infrastructure, a channel is needed to collect information on essential elements, such as available personnel, equipment, facilities, and reagent manufacturers.

If a crisis is expected, expansion of testing capacity should begin as soon as possible. The testing demand for a crisis might grow larger and faster than that required to respond to an epidemic. For new pathogens, a lack of information on transmission routes and fatality rates can cause excessive psychological anxiety among the population, which can directly lead to the demand for testing. During the South Korea MERS outbreak in 2015, a total of 44,768 MERS tests were conducted, from the first case reported on May 19, 2015, through December 31, 2015. A total of 186 patients were confirmed, which means ≈241 tests were conducted to identify each patient (*5*). During the early stages of the COVID-19 pandemic beginning on January 20, 2020 (when the first case was detected in South Korea), to the end of June 2020, a total of 2,460,389 tests were performed, identifying 12,799 confirmed cases. In total, ≈192 tests were needed to detect each patient. Expanding testing capabilities requires cooperation among various responding entities. Large-scale testing requires a large number of diagnostic test reagents, testing agencies, and workforce, and a support and management system is required to maintain testing accuracy, especially during large-scale testing. It is difficult to provide all of those elements within a short period. Therefore, the time to prepare for those factors must be considered when deciding to expand the testing capacity, and testing capacity expansion should begin well before the actual need for large-scale testing. After WHO declared a Zika virus PHEIC in 2016, KDCA decided to expand its testing capacity to include the private sector. It took ≈60 days to secure diagnostic reagents for Zika virus through the EUA, recruit testing agencies from private medical institutions, and expand testing capabilities in the private sector (*6*). For COVID-19 in 2020, the duration for those efforts was markedly reduced, to 10 days (Figure 2). The COVID-19 test was PCR based and relied on the same amplification principle as the Zika virus test. Detailed procedures and methods from the EUA and the designation of a testing agency for the Zika response could be used for COVID-19, making it possible to respond within 10 days. Therefore, if previous experience cannot be used because the outbreak is cause by a completely new pathogen, more time might be needed to expand testing capabilities. In addition, if no companies in South Korea were directly involved in development and production of reagents, and reliance on imports was necessary, it would have taken substantially more time to respond. In summary, demand for infectious disease testing is increasing rapidly, and an absolute time needed to expand testing capacity does exist. Therefore, when a crisis is predicted, the expansion of testing capacity should begin without delay.

Another consideration is to ensure test accuracy. Tests are conducted to reduce uncertainty by quickly identifying infected persons, enabling necessary measures such as isolation and treatment. Therefore, further confusion might occur if the accuracy of the test is low. Frequent retesting because of a lack of trust in the test results can reduce testing capacity, and incorrect quarantine measures can cause harm to persons or accelerate the spread of infectious diseases if an infected person is missed. During the early stages of response to a new pathogen, no other control measures exist, such as vaccines or treatments, and control policies are mainly used in accordance with test results; therefore, reliability of the diagnostic tests is even more critical. If the test results cannot be trusted, it is challenging to expect public trust and cooperation in subsequent quarantine measures. The accuracy of the test is essential, but the possibility of errors in testing for new pathogens might be higher than for pathogens that have been continuously tested. When testing for pathogens has never been performed before, difficulties arise during the testing process and interpretation of results. Therefore, tools are required to ensure testing accuracy. In response to COVID-19, all testing agencies in South Korea must complete training and participate in external quality assessment by using artificial samples to check the agency’s testing ability (*8*). In addition, guidelines for COVID-19 testing were continuously distributed in response to situational changes. Those guidelines included considerations for COVID-19 testing in newly expanded laboratories and covered testing methods, specimen selection, biosafety recommendations, and specimen transport. Furthermore, practical expert opinions were incorporated to ensure accurate testing in real-world scenarios, including sample pretreatment, retesting, follow-up testing, and reagent selection (*9*–*11*). KDCA and private experts visited the agencies where testing errors occurred to identify factors responsible for the errors and to provide guidance on how to address those errors. Cooperation with private experts is essential to provide guidance and ensure test accuracy. During a crisis caused by a new pathogen, it is necessary to identify various events that occur in the field and synthesize new information in real time to suggest appropriate solutions. However, those tasks are limited by government capabilities by themselves. With the strong cooperation of several experts, South Korea rapidly conducted education and external quality assessment during the early stage of the COVID-19 pandemic, and we continuously monitored the situation. We managed the on-site testing situation despite the long-term response to COVID-19. For such a robust cooperative system to work in times of crises, it is essential to build trust through cooperation and regular exchanges during times of stability.

## Conclusion

The ability to conduct sufficient testing, especially during an infectious disease crisis, is crucial in fighting infectious diseases. The widespread use of new strategies comes with both responsibilities and risks. In response to COVID-19, efforts have been made to maximize the benefits and minimize the risks of using new strategies, such as identifying the best test reagents by evaluating and managing the institutions that use them. In South Korea, those efforts were quickly accomplished. However, each country faces unique circumstances and challenges in preparing for diagnostic laboratory and related infrastructure expansion, making it difficult to directly apply the approach used in South Korea. Nevertheless, South Korea’s experience will inform future considerations involved in expanding laboratory systems during emerging infectious disease outbreaks.

Diagnostic testing technologies are developing rapidly. Various analytical methods that require high-level expertise, such as bioinformatics and metagenomics, are being developed, and simple, miniaturized, and modularized test products are also being developed by using new materials and technology. Technologies that have never been used before might need to be applied in response to pathogens. Therefore, a supplementary measure to secure large-scale testing capabilities more rapidly and further reduce new risks was needed, according to our experience. In preparation for the next pandemic, KDCA formed a public–private joint evaluation group that could evaluate various diagnostic test methods or products. Through preemptive evaluation, we want to secure information in advance, such as the performance and usability of various testing methods, and enhance national evaluation skills in various environments so that diagnostic products can be evaluated appropriately, even during a crisis. In addition, KDCA plans to designate institutions that have secured the infrastructure necessary for infectious disease testing. Usually, personnel from designated institutions participate in training to respond to infectious diseases by using the KDCA to learn new testing technologies; private medical institutions can prioritize testing for new pathogens in the event of infectious disease transmission. The KDCA in South Korea provides a laboratory diagnostic system that ensures safety for the timely introduction of novel strategies used to prevent the spread of new pathogens.
